# Effects of the cervical headgear in growing Angle Class II malocclusion patients: a prospective study

**DOI:** 10.1590/2177-6709.25.2.025-031.oar

**Published:** 2020

**Authors:** Anderson Jaña Rosa, Rizomar Ramos do Nascimento, José Nelson Mucha, Oswaldo de Vasconcellos Vilella

**Affiliations:** 1 Universidade Federal Fluminense, Faculdade de Odontologia, Departamento de Ortodontia (Niterói/RJ, Brazil).

**Keywords:** Extraoral traction appliances, Malocclusion, Angle Class II, Tooth movement techniques, Orthodontics

## Abstract

**Objective:**

Evaluate dental and skeletal changes resulting from the exclusive use of the cervical headgear for 15 ± 4 months in the treatment of patients with Class II division 1 malocclusion.

**Methods:**

Differences between the beginning (T_1_) and immediately after the end of the therapy (T_2_) with the cervical headgear in growing patients (Experimental Group, EG, n = 23) were examined and compared, during compatible periods, with those presented by a group of untreated individuals (Control Group, CG, n =22) with similar malocclusions and chronological age. The cephalometric variables evaluated were: ANB, GoGn.SN, AO-BO, S'-ANS, S'-A, S'-B, S'-Pog and S'-U6 (maxillary first molar). The Shapiro-Wilk and Levene tests were used to evaluate the results.

**Results:**

Significant differences were found relative to the ANB, S'-U6, AO-BO, S'-ANS, S'-A, S'-B and S'-Pog variables between T_1_ and T_2_ when comparing both groups. No statistically significant variation was found regarding the GoGn.SN angle.

**Conclusions:**

The use of cervical headgear promoted distal movement of the maxillary first molars and restricted the anterior displacement of the maxilla, without significantly affecting the GoGn.SN angle.

## RESUMO

**Objetivo:**

Avaliar as alterações dentárias e esqueléticas decorrentes do uso exclusivo do aparelho extrabucal durante 15 ± 4 meses para tratamento de pacientes com má oclusão de Classe II divisão 1 (Grupo Experimental, GE).

**Métodos:**

As diferenças entre o início (T_1_) e imediatamente após o término da terapia (T_2_) com o aparelho extrabucal de tração cervical (Grupo Experimental, GE, n = 23) foram comparadas àquelas apresentadas por um grupo composto por indivíduos não tratados (Grupo Controle, GC, n = 22), com má oclusão e faixa etária cronológica compatíveis. As variáveis cefalométricas avaliadas foram: ANB, GoGn.SN, AO-BO, S’-ENA, S’-A, S’-B, S’-Pog e S’-U6 (primeiro molar superior). Os testes de Shapiro-Wilk e Levene foram aplicados para avaliar os resultados.

**Resultados:**

Diferenças significativas entre T_1_ e T_2_ foram encontradas para as variáveis ANB, S’-U6, AO-BO, S’-ENA, S’-A, S’-B e S’-Pog, quando comparados os dois grupos. Nenhuma diferença estatisticamente significativa foi encontrada em relação ao ângulo GoGn.SN.

**Conclusão:**

O uso do aparelho extrabucal com tração cervical promoveu movimento para distal do primeiro molar superior e restringiu o deslocamento anterior da maxila, sem afetar significativamente o ângulo GoGn.SN.

## INTRODUCTION

The use of extraoral cervical traction appliance was one of the most common strategies to correct Angle Class II malocclusion in growing patients.^1^ ^-^ ^4^ However, the use of cervical headgear has decreased over the last decades^5^ due to the development of more aesthetic procedures, such as those involving fixed or removable orthopedic appliances that rely less on patient compliance.^5^ ^-^ ^7^

Despite the decline of its use, the effects of the cervical headgear in the Class II malocclusion treatment, combining skeletal and dentoalveolar changes, have been confirmed by several authors.^8^ ^-^ ^10^ The extraoral cervical appliance placed on the maxillary first molar promotes changes in the anteroposterior and vertical directions, which are reflected by the changes in the morphological characteristics of the alveolar processes and basal bones.^4^ ^,^ ^9^ ^,^ ^11^ ^,^ ^12^ The ideal moment to correct maxillary protrusion seems to be during the mixed dentition phase, just before the growth spurt. Therefore, a well-timed and adequate intervention at this stage may reduce the anteroposterior discrepancy between the jaws.^1^ ^,^ ^11^

In this approach, the patient's facial growth pattern and desired treatment outcome determine the direction of the pull, as well as the extent and angulation of the outer bow. The extraoral cervical traction device has greater acceptability when compared to high- or straight-pull traction appliances. It is often recommended for patients with hypodivergent or mesodivergent skeletal pattern.^2^ ^,^ ^13^ ^,^ ^14^

Several authors^1-12^ have investigated the influence of extraoral appliance with cervical traction on the dentofacial complex. The distal movement of the first maxillary molars and the change in the inclination of the mandibular plane are among the most discussed and contradictory aspects of this treatment.

However, few researchers have focused efforts on evaluating the exclusive action of the extraoral cervical traction on the maxillary first molars and related structures. In previous studies, the headgear was combined with fixed or functional appliances. Some authors have recommended the use of the cervical headgear during the retention phase, while others have performed measurements using radiographs taken during the posttreatment phase.^15^ ^,^ ^16^ After a long span of time, the growth pattern observed before treatment may return or mask the effects that would be observed in studies conducted over a short period of time.^17^

This study aimed at evaluating the magnitude of skeletal changes of the exclusive use of extraoral cervical traction in patients with Angle Class II malocclusion, in addition to its effects on the maxillary first molars.

## MATERIAL AND METHODS

The present study was approved by the Fluminense Federal University Research Ethics Committee (protocol number: 924304). The sample size was calculated using the formula described by Pandis^32^ in a previous pilot project: for the results of the study to have statistical representativeness, each group should have at least 22 subjects. The study had 80% power to detect differences between the experimental and control groups at 5% significance level.

**Figure 1:**
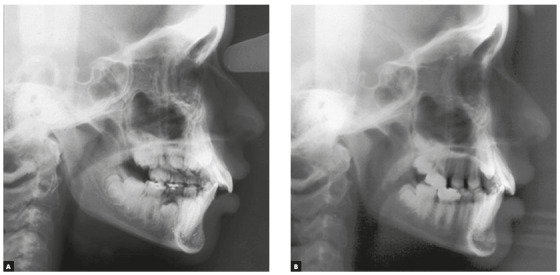
A) Initial radiograph, at the beginning of the treatment. B) Radiograph obtained after cervical headgear treatment.

The patients who composed the experimental group (EG) were consecutively recruited at the Orthodontics Clinic of the *Universidade Federal Fluminense*. The selection criteria was the presence of well-defined Class II malocclusion, in which the vestibular cusp of the first maxillary molar occludes in the mesiobuccal sulcus of the first lower molar. These patients were treated exclusively with extraoral cervical traction until the Class I molar relationship or an overcorrection was attained (Fig 1). Low-quality radiographs that would compromise the examination of the relevant anatomical markers were excluded. Therefore, the EG was composed by 23 patients (10 males and 13 females). All individuals were treated with cervical headgear at the same institution.

Forty-six cephalometric tracings were evaluated and measured. Initial radiographs were obtained at the beginning of treatment (T_1_). The average age at T_1_ was 10 years and 8 months (± 1 year). Final radiographs were obtained immediately after the end of the treatment with the extraoral appliance. The average age at T_2_ was 12 years (± 1 year). In the EG, the minimum and maximum ages at T_1_ were 8 years and 12 years and 7 months, respectively. In the T_2_, the minimum age was 9 years and 3 months, and the maximum age was 13 years and 10 months. The treatment duration was 15 ± 4 months on average. All the cephalometric examinations were performed at the same radiographic center and traced by the same operator.

The cervical headgear consisted of an inner bow soldered in the midline to an outer bow, with diameters measuring 0.050” and 0.075”, respectively. The inner bow was bent with stop bends directed occlusally, mesial to the first molar band tubes, to grant approximately 4 mm between the maxillary incisors and the extraoral arch. The outer bow was made parallel to the inner bow and was 2.5 cm wider than the latter. Elastics were set from the outer bow to a cervical pad on each side, connecting and tying them together. The force applied by the elastics was 350-450g. The patients were recommended to use the cervical headgear 14 to 16 hours a day, replacing the elastics every week.

The Control Group (CG) consisted of 44 lateral cephalograms obtained from 22 Canadian subjects (10 male and 12 female), with Angle Class II malocclusion. They were followed-up at the Burlington Research Centre, University of Toronto, Canada, for an average time frame of 16 ± 6 months, without any kind of orthodontic appliance. The initial mean age (T_1_) was 10 years and 8 months ± 1 year and 1 month, and the average age at the end of follow-up (T_2_) was 12 years ± 1 year and 2 months. The participants in this group were matched to the EG according to the chronological age. The radiographic magnifications and distortions were previously corrected, in a similar way to that described by Thompson and Popovich.^18^

**Figure 2:**
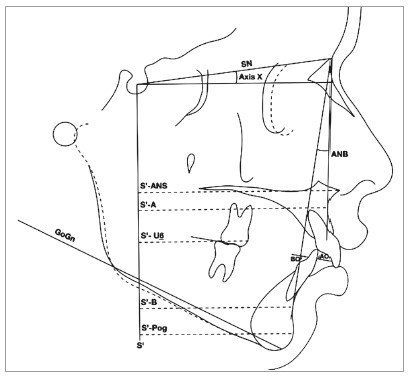
Cephalogram with the angular and linear measurements used in the study.

Points, lines, and measures used in cephalometric analysis to assess the effects of cervical headgear compared to the CG are described in Table 1. The linear measurements were obtained with a digital caliper (Starrett, Itu, São Paulo, Brazil), with an accuracy of 0.01mm, and the angular measurements were made with a protractor (Acrimet, São Bernardo do Campo, São Paulo, Brazil). The enlargement and distortion of the mid-sagittal plane was 8%. The values were converted and organized in spread sheets (Excel 2016, version 15.28, Microsoft Office Corp., Santa Rosa, California, USA). A cephalogram with linear and angular measurements is shown in Figure 2.

**Table 1:** Points, lines and variables utilized.

Points and lines	Definition
Line SN	Line from point Sella (S) to point Nasion (N)
Axis X	Orientation axis: Axis from point S, forming an angle of 7 degrees with line SN
Line S’	Line from Point S, perpendicular to Axis X
Line NA	Line from N to point A
Line NB	Line from N to point B
Point U6	Most mesial point of maxillary first molar crown
Variables	Definition
ANB (degrees)	Angle formed by intersection of NA and NB lines
GoGn.SN (degrees)	Mandibular plane angle
AO-BO (mm)	Distance between AO and BO points
S’-ANS (mm)	Linear distance and perpendicular from line S’ to point ANS
S’- A (mm)	Linear distance and perpendicular from line S’ to point A
S’-U6 (mm)	Linear distance and perpendicular from line S’ to point U6
S’-B (mm)	Linear distance perpendicular from line S’ to point B
S’-Pog (mm)	Linear distance perpendicular from line S’ to point Pog

To assess the intra- and inter-examiners reliability, eight cephalograms were randomly selected in the experimental group, and the measurements were repeated seven days after the initial measurements. A second examiner independently performed the same measurements. 

The paired *t* test was applied using the Quick Calcs Graph Pad software (2013 version), available at *www.graphpad.com/quickcalcs*. The Shapiro-Wilk test defined non-parametric tests for intragroup comparison (Wilcoxon test), and intergroup comparisons (Mann-Whitney). The Levene test was used to evaluate the homogeneity of variances between the two groups. Statistical analyzes were performed using SPSS software, version 20.0 (IBM Corp., Illinois, Chicago, USA). A probability level of 5% was adopted (*p*< 0.05).

## RESULTS

The paired *t* test indicated no statistically significant differences between intra- and inter-examiners measurements. The intraexaminer method error varied from *p*= 1.00 (AO-BO) to *p*= 0.92 (GoGn.SN). The interexaminers error ranged from *p*= 1.00 (AO-BO) to *p*= 0.77 (S'-ANS). Therefore, the error of the method was considered of no importance in this study. 

Levene’s test found that the subjects in the EG presented significantly higher maxillomandibular discrepancy ANB (F = 0.076, *p*= 0.000) and AO-BO (F = 0.011, *p*= 0.011) than individuals of the CG at T_1_. Other variables did not differ significantly.

The mean values of the EG and CG at T_1_ and T_2_, and the differences between these means and between the groups are available in Table 2.

**Table 2:** Mean values in the experimental (EG) and control groups (CG) at T1 and T2, standard deviation (SD), Standard Error Mean (SEM), Wilcoxon test to compare the differences between T1 and T2, and Mann-Whitney test between EG x CG.

		EG (n=23)				CG (n=22)				EG x CG			
Variable	Phase	mean	SD	SEM	T2-T1	p-value	mean	SD	SEM	T2-T1	p-valor	Difference	p-valor
ANB	T1	6.3	1.29	0.268	-1.5	0.000*	4.14	1.34	0.285	0.02	0.858^ns^	-1.52	0.000*
	T2	4.8	1.76	0.366			4.16	1.45	0.309				
AO-BO	T1	3	1.97	0.411	-0.93	0.002*	1.36	2.18	0.466	0.39	0.138^ns^	-1.32	0.001*
	T2	2.07	2.1	0.437			1.75	2.55	0.544				
S’-ANS	T1	71.85	5.04	1.051	-0.11	0.819^ns^	70	4.12	0.879	1.43	0.001*	-1.54	0.013*
	T2	71.74	5.96	1.242			71.43	3.92	0.836				
S’-A	T1	67.33	5.97	1.245	-0.76	0.064^ns^	64.98	4.1	0.873	1.55	0.000*	-2.31	0.000*
	T2	66.57	6.67	1.39			66.52	4.29	0.916				
S’-B	T1	55.7	8.5	1.772	0.57	0.115^ns^	55.52	6.02	1.285	1.84	0.000*	-1.27	0.012*
	T2	56.26	9.05	1.886			57.36	6.25	1.33				
S’-Pog	T1	55.09	8.98	1.873	0.61	0.083^ns^	55.89	6.61	1.41	2.16	0.000*	-1.55	0.003*
	T2	55.7	9.2	1.919			58.05	6.72	1.432				
S’-U6	T1	39.41	6.1	1.271	-5.15	0.000*	38.14	3.9	0.832	2.25	0.000*	-7.4	0.000*
	T2	34.26	7.03	1.466			40.39	4.13	0.88				
GoGn.SN	T1	37.41	6.26	1.306	-0.04	0.894^ns^	35.93	5.92	1.262	-0.2	0.574^ns^	0.16	0.740^ns^
	T2	37.37	6.16	1.285			35.73	5.44	1.159				

* = p < 0.05; ns = Non-significant.

Comparing the two groups (EG x CG), all evaluated variables presented statistically significant differences, except the GoGn.SN angle.

In the EG, all subjects presented significant differences for the variable S'-U6, from T_1_ to T_2_, with an average change of -5.15 mm (*p*= 0.000). The maxillomandibular discrepancy was significantly reduced in the EG, with the ANB angle mean changed by -1.50^o^ (*p*= 0.000), and the linear measure AO-BO mean changed by -0.93 mm (*p*= 0.002).

In the CG, ANB and AO-BO values increased without statistical significance (*p*= 0.858 and *p*= 0.138, respectively), and the variable S'-U6 increased by 2.25 mm (*p*= 0.000) between T_1_ and T_2_. Significant differences were also observed for S'-ANS (*p*= 0.001), S'-A (*p*= 0.000), S'-B (*p*= 0.000) and S'-Pog (*p*= 0.000).

## DISCUSSION

In the present research, the subjects in the EG utilized a cervical traction on the maxillary first permanent molars without any other orthodontic appliance. The effects on these teeth, on the maxilla and mandible were evaluated.

The most discussed and controversial points of treatment with cervical headgear are the orthopedic effects on the maxilla, the movements of the maxillary first molars, and changes in the mandibular plane inclination.^3^ ^,^ ^8^ ^,^ ^9^ ^,^ ^11^ ^,^ ^12^ ^,^ ^19^ ^-^ ^22^

Comparative studies on these changes are limited by the difficulty in obtaining an adequate control group (CG).^10^ ^,^ ^15^ ^,^ ^19^ ^,^ ^23^ The present study used a CG composed by untreated individuals with malocclusions, observation time and chronological ages similar to those of the EG. The treatment response of the EG individuals showed that the therapy was carried out within the pubertal growth spurt. Thus, significant changes at this stage were observed, even over a period of only 15 months.

A significant contrast in the displacement direction of the maxilla was observed between the Experimental and Control Groups. The EG showed a backward displacement of the variables S'-A and S'-ANS between T_1_ and T_2_, whereas in the CG this displacement occurred forward. This finding suggests that the cervical headgear alone promoted a distal or restrictive orthopedic effect on maxillary advancement. These results are consistent with several studies that described the backward displacement of point A in subjects treated with cervical headgear to correct Class II malocclusion in the late mixed dentition, as a result of backward movement of the maxilla.^4^ ^,^ ^8^ ^-^ ^12^ ^,^ ^15^ ^,^ ^16^ ^,^ ^19^ ^,^ ^21^ ^,^ ^24^ ^-^ ^26^ Some authors have demonstrated the effectiveness of cervical headgear on deeper structures such as the pterygomaxillary fissure.^10^ ^,^ ^16^

Statistically significant variations between groups were also observed for the sagittal displacement of the maxillary first molars to mesial in the CG (S'-U6 = 2.25 ± 1.25 mm), while in the EG there was remarkable distal displacement (-5.15 ± 3.41 mm). Some studies showed similar results to those presented herein,^2^ ^,^ ^3^ ^,^ ^20^ ^,^ ^27^ ^-^ ^30^ while others verified mesial movement of the maxillary molars, even after undergoing therapy with an extraoral appliance.^10^ ^,^ ^13^ ^,^ ^20^ In these studies, the radiographs were taken at T_2_ at three^15^, six^25^ or 42 months^10^ after the end of the complete treatment with braces on the other teeth, or after the retention phase. 

It can be assumed that it is possible to correct Class II malocclusion with the use of a cervical headgear for 12 to 15 months. If the treatment is completed with fixed appliances for a few more months or years, growth/anterior displacement of both the maxilla and the mandible is likely to occur. This growth after the use of a cervical headgear will mask the distal effects on the molar,^8^ ^,^ ^26^ due to the forward maxillary displacement. For this reason, studies to evaluate the effects of molar distalization should be conducted over short periods of time and with the exclusive use of a cervical headgear, according to the design of the present study.

As the values observed for S'-U6, S'-A and S'-ANS decreased between T_1_ and T_2_, it can be inferred that the extraoral force acts with greater intensity on the first molars, reducing its orthopedic influence gradually, and it should also be considered that no brackets were installed on the anterior maxillary teeth.

Several studies have demonstrated an increase in the mandibular plane angle as a result of extrusion and tipping of the maxillary molars with the use of headgear with cervical traction.^8^ ^,^ ^12^ ^,^ ^23^ ^,^ ^29^ ^,^ ^31^ Elements that may be considered determinant of this effect are the inclination and the length of the outer bow in relation to the inner bow.^31^ In the current study design, the forces passed below the center of resistance (CR) of the maxillary first molars, causing distal tipping of these teeth. The purpose was to improve anchorage to retract the anterior dental segment. When the linear distance from S’ line to the CR of the first maxillary molar was measured, the values were: at T_1_, S’-CR = 34.96 ± 5.73 mm; at T_2_, S’-CR = 31.48 ± 3.48 mm. So, the difference between the two moments was 3.48 mm.

The EG, as well as the CG, exhibited a decrease in the inclination of the mandibular plane (GoGn.SN) between T_1_ and T_2_, despite the non-significant differences. This is consistent with other studies that considered this finding clinically irrelevant or did not detect changes following the use of mechanical appliances.^11^ ^,^ ^19^ ^,^ ^21^ ^,^ ^22^ Favorable progress in the condylar and alveolar regions have been described as possible factors that allow treatment without changes in the mandibular plane,^12^ or with only clinically insignificant changes.^2^ ^,^ ^11^ The time of use and the force employed may also have contributed to the present results, partly being compensated by the facial growth pattern of the subjects of this sample.

The CG showed a significant increase in the variables S'-B (*p*= 0.000) and S'-Pog (*p*= 0.000) from T_1_ to T_2_. Although the EG also showed an increase in these variables, it was not statistically significant (*p*= 0.115 and *p*= 0.083, respectively). Some authors^11^ ^,^ ^23^ reported the extrusion of the first permanent molars as one of the side effects of the cervical traction, with mandibular clockwise rotation.^3^ ^,^ ^23^ As a result, the landmarks B and Pog tend to take a more low and posterior position.^9^ However, the prescribed treatment utilized by the EG (including the design of the cervical headgear) was effective, without significant adverse effects on the mandibular plane angle (GoGn.SN), corroborating other findings.^11^

Comparison between the two groups suggests remarkable improvement in the maxillomandibular relationship among the treated patients of the EG. The ANB and AO-BO values decreased (*p*= 0.000 and *p*= 0.001, respectively) in the EG, differing significantly from the untreated CG individuals. This reduction implies the orthopedic action of headgear with cervical traction on the maxilla, even when supported only in the maxillary first molars. 

One of the research’s limitations was the impossibility of selecting a control group with the same ethnic origin and similar characteristics to those of EG, due to ethical restrictions. The alternative was to use a preexisting CG, although the means of the initial values of the ANB and AO-BO variables differ between the groups. Another limitation was the absence of hand and wrist radiographs, which prevented a fine comparison of the subjects from groups EG and CG regarding their growth and development stages by the skeletal maturity alongside the chronological ages. These limitations are expected to be overcome in future studies.

## CONCLUSIONS

In the current study, cervical headgear led to: restriction of the forward maxillary displacement; distal movement of the maxillary first molars; sagittal discrepancy reduction in the maxillomandibular relationship, with consequent correction of the Class II malocclusion; and no increase in the inclination of the mandibular plane.

## References

[1] Weislander L (1975). Early or late cervical traction therapy of Class II malocclusion in the mixed dentition. Am J Orthod.

[2] Farret MM, Lima EM, Araújo VP, Rizzatto SMD, Menezes LM, Grossi ML (2008). Molar changes with cervical headgear alone or in combination with rapid maxillary expansion. Angle Orthod.

[3] Henriques FP, Janson G, Henriques JFC, Pupulim DC (2015). Effects of cervical headgear appliance a systematic review. Dental Press J Orthod.

[4] Kirjavainen M, Hurmerinta K, Kirjavainen T (2007). Facial profile changes in early Class II correction with cervical headgear. Angle Orthod.

[5] Tüfekçi E, Allen SB, Best AM, Lindauer SJ (2016). Current trends in headgear use for the treatment of class II malocclusions. Angle Orthod.

[6] Almuzian M, Alharbi F, McIntyre G (2016). Extra-oral appliances in orthodontic treatment. Dent Update.

[7] Cassidy SE, Jackson SR, Turpin DL, Ramsay DS, Spiekerman C, Huang GJ (2014). Classification and treatment of Class II subdivision malocclusions. Am J Orthod Dentofacial Orthop.

[8] Angelieri F, De Almeida RR, Janson G, Castanha Henriques JF, Pinzan A (2008). Comparison of the effects produced by headgear and pendulum appliances followed by fixed orthodontic treatment. Eur J Orthod.

[9] Polat-Ozsoy O, Gokcelik A, Güngör-Acar A, Kircelli BH (2008). Soft tissue profile after distal molar movement with a pendulum K-loop appliance versus cervical headgear. Angle Orthod.

[10] Weislander L (1963). The effect of orthodontic treatment on the concurrent development of the craniofacial complex. Am J Orthod.

[11] Lima RMA, Lima AL, Ruellas ACO (2003). Mandibular changes in skeletal Class II patients treated with Kloehn cervical headgear. Am J Orthod Dentofacial Orthop.

[12] Phan XL, Schneider BJ, Sadowsky C, Begole EA (2004). Effects of orthodontic treatment on mandibular rotation and displacement in Angle Class II division 1 malocclusions. Angle Orthod.

[13] Casaccia GR, Gomes JC, Squeff LR, Penedo ND, Elias CN, Gouvêa JP (2010). Analysis of initial movement of maxillary molars submitted to extrabucal forces: a 3D study. Dental Press J Orthod.

[14] Ibitayo AO, Pangrazio-Kulbersh V, Berger J, Bayirli B (2011). Dentoskeletal effects of functional appliances vs bimaxillary surgery in hyperdivergent Class II patients. Angle Orthod.

[15] Mossaz CF, Byloff FK, Kiliaridis S (2007). Cervical headgear vs pendulum appliance for the treatment of moderate skeletal Class II malocclusion. Am J Orthod Dentofacial Orthop.

[16] Piva LM, Brito HHA, Leite HR, O'reilly M (2005). Effects of cervical headgear and fixed appliances on the space available for maxillary second molars. Am J Orthod Dentofacial Orthop.

[17] Gandini MREAS, Gandini LG, Martins JCR, Del Santo M (2001). Effects of cervical headgear and edgewise appliances on growing patients. Am J Orthod Dentofacial Orthop.

[18] Thompson GW, Popovich F (1977). A longitudinal evaluation of the Burlington Growth Centre Data. J Dent Res.

[19] Antonarakis GS, Kiliaridis S (2015). Treating Class II malocclusion in children Vertical skeletal effects of high-pull or low-pull headgear during comprehensive orthodontic treatment and retention. Orthod Craniofac Res.

[20] de Lira ALS, Izquierdo A, Prado S, Nojima M, Maia L (2012). Anteroposterior dentoalveolar effects with cervical headgear and pendulum appliance a systematic review. Braz J Oral Sci.

[21] Lione R, Franchi L, Laganà G, Cozza P (2015). Effects of cervical headgear and pendulum appliance on vertical dimension in growing subjects: a retrospective controlled clinical trial. Eur J Orthod.

[22] Tamburús VS, Pereira JS, Siqueira VCV, Tamburús WL (2011). Treatment effects on Class II division 1 high angle patients treated according to the Bioprogressive therapy (cervical headgear and lower utility arch), with emphasis on vertical control. Dental Press J Orthod.

[23] Brown P (1978). A cephalometric evaluation of high-pull molar headgear and face-bow neck strap therapy. Am J Orthod.

[24] Alió-Sanz J, Iglesias-Conde C, Lorenzo-Pernía J, Iglesias-Linares A, Mendoza-Mendoza A, Solano-Reina E (2012). Effects on the maxilla and cranial base caused by cervical headgear: a longitudinal study. Med Oral Patol Oral Cir Bucal.

[25] Baccetti T, Franchi L, Stahl F (2009). Comparison of 2 comprehensive Class II treatment protocols including the bonded Herbst and headgear appliances a double-blind study of consecutively treated patients at puberty. Am J Orthod Dentofacial Orthop.

[26] Almeida-Pedrin RR, Henriques JF, Almeida RR, Almeida MR, Mcnamara JA (2009). Effects of the pendulum appliance, cervical headgear, and 2 premolar extractions followed by fixed appliances in patients with Class II malocclusion. Am J Orthod Dentofacial Orthop.

[27] Abed Y, Brin I (2010). Early headgear effect on the eruption pattern of maxillary second molars. Angle Orthod.

[28] Gkantidis N, Halazonetis DJ, Alexandropoulos E, Haralabakis NB (2011). Treatment strategies for patients with hyperdivergent Class II Division 1 malocclusion: Is vertical dimension affected. Am J Orthod Dentofacial Orthop.

[29] Maruo I, Maruo H, Saga A, de Oliveira D, Argenta M, Tanaka O (2016). Tridimensional finite element analysis of teeth movement induced by different headgear forces. Prog Orthod.

[30] Melsen B, Dalstra M (2003). Distal molar movement with Kloehn headgear Is it stable?. Am J Orthod Dentofacial Orthop.

[31] Merrifield LL, Cross JJ (1970). Directional forces. Am J Orthod.

[32] Pandis N, Polychronopoulou A, Eliades T (2011). Effects of cervical headgear and pendulum appliance on vertical dimension in growing subjects trial designs. Am J Orthod Dentofacial Orthop.

